# Supplementary material for: A Comprehensive Assessment of Ultraviolet-Radiation-Induced Mutations in Flammulina filiformis Using Whole-Genome Resequencing
Source: J Fungi (Basel). 2024 Mar 20;10(3):228. doi: 10.3390/jof10030228 (PMC10971301; doi:10.3390/jof10030228)
Supplement: Supplementary file 1 [file jof-10-00228-s001.zip › Supplementary Material S8/KEGG annotation/out/64381550635650.os/KO/out_map/map05031.html]

KEGG PATHWAY: Amphetamine addiction - Reference pathway


|  |  |
| --- | --- |
| **Amphetamine addiction - Reference pathway** |  |

[
Pathway menu
| Organism menu
| Pathway entry
| Show description
| User data mapping
]

Amphetamine is a psychostimulant drug that exerts persistent addictive effects. Most addictive drugs increase extracellular concentrations of dopamine (DA) in nucleus accumbens (NAc) and medial prefrontal cortex (mPFC), projection areas of mesocorticolimbic DA neurons and key components of the "brain reward circuit". Amphetamine achieves this elevation in extracellular levels of DA by promoting efflux from synaptic terminals. Acute administration of amphetamine induces phosphorylation of cAMP response element-binding protein (CREB) and expression of a number of immediate early genes (IEGs), such as c-fos. The IEGs is likely to initiate downstream molecular events, which may have important roles in the induction and maintenance of addictive states. Chronic exposure to amphetamine induces a unique transcription factor delta FosB, which plays an essential role in long-term adaptive changes in the brain.

|  |  |
| --- | --- |
| Reference pathway | 100% |
